# An Online Tool for Nurse Triage to Evaluate Risk for Acute Coronary Syndrome at Emergency Department

**DOI:** 10.1155/2015/413047

**Published:** 2015-04-02

**Authors:** Yuwares Sittichanbuncha, Patchaya Sanpha-asa, Theerayut Thongkrau, Chaiyapon Keeratikasikorn, Noppadol Aekphachaisawat, Kittisak Sawanyawisuth

**Affiliations:** ^1^Emergency Medicine Department, Faculty of Medicine, Ramathibodi Hospital, Mahidol University, Bangkok 10400, Thailand; ^2^Department of Computer Sciences, Faculty of Sciences, Khon Kaen University, Khon Kaen 40002, Thailand; ^3^Central Library, Silpakorn University, Bangkok 10200, Thailand; ^4^Department of Medicine, Faculty of Medicine, Khon Kaen University, Khon Kaen 40002, Thailand; ^5^Research Center in Back, Neck, Other Joint Pain and Human Performance (BNOJPH), Khon Kaen University, Khon Kaen 40002, Thailand

## Abstract

*Background*. To differentiate acute coronary syndrome (ACS) from other causes in patients presenting with chest pain at the emergency department (ED) is crucial and can be performed by the nurse triage. We evaluated the effectiveness of the ED nurse triage for ACS of the tertiary care hospital. *Methods*. We retrospectively enrolled consecutive patients who were identified as ACS at risk patients by the ED nurse triage. Patients were categorized as ACS and non-ACS group by the final diagnosis. Multivariate logistic analysis was used to predict factors associated with ACS. An online model predictive of ACS for the ED nurse triage was constructed. *Results*. There were 175 patients who met the study criteria. Of those, 28 patients (16.0%) were diagnosed with ACS. Patients with diabetes, patients with previous history of CAD, and those who had at least one character of ACS chest pain were independently associated with having ACS by multivariate logistic regression. The adjusted odds ratios (95% confidence interval) were 4.220 (1.445, 12.327), 3.333 (1.040, 10.684), and 12.539 (3.876, 40.567), respectively. *Conclusions*. The effectiveness of the ED nurse triage for ACS was 16%. The online tool is available for the ED triage nurse to evaluate risk of ACS in individuals.

## 1. Background

Chest pain is the second common presentation at the emergency department (ED) [1]. There are several causes of chest pain such as acute coronary syndrome (ACS), pleuritic chest pain, or costochondritis. ACS has high morbidity and mortality rate among other causes of chest pain. It comprises two conditions: acute myocardial infarction (AMI) and unstable angina.

Coronary artery disease (CAD) is the leading cause of death in Thailand and worldwide [2, 3]. At the ED, prompt diagnosis and treatment are crucial to reduce morbidity and mortality from ACS [3]. Nurse triage at the ED has been shown to identify individuals with ACS [4, 5]. Using flowchart by the nurse triage is effective to exclude ACS [5]. Factors associated with non-ACS were age less than 40 years, no diabetes, no previous history of CAD, and no typical chest pain [5].

Ramathibodi Hospital is a tertiary and university hospital located in Bangkok, Thailand. Nursing triage at the ED for ACS has been established and aims to correctly identify ACS patients. Patients presenting with chest pain are firstly evaluated by the ED nurses. Those patients who are at risk for ACS were evaluated by the triage nurse; ACS fast track will be activated. The effectiveness of the nurse triage at the ED has never been evaluated. We aimed to study the correlation of nurse triage for ACS and final diagnosis of each patient and what factors are suggestive for ACS and create an online tool for nurse triage at the ED to identify patients with ACS.

## 2. Methods

Consecutive patients who were identified as ACS suspected by nursing triage at the ED, Ramathibodi Hospital, Mahidol University, Thailand, were enrolled. The study period was between December 1, 2006, and June 30, 2007. The final diagnosis of each patient was made by an attending physician at discharge.

Charts of all patients enrolled by ACS nursing triage at the ED were retrospectively reviewed. The study variables included baseline characteristics, comorbid diseases, characters of chest pain, risk factors for CAD, and laboratory results. Chest pain characters of ACS or angina chest pain was one of these following features: chest tightness, squeeze pain in the chest, pain referred to shoulder, arm, or mandible, difficulty in breathing, tachypnea, dull aching pain at epigastrium, presence of sweating, or unexplained fatigue, palpitation, syncope, or pain not relieved by sublingual nitroglycerin. Risk factors for CAD were older age, obesity, dyslipidemia, smoking, hypertension, diabetes, male gender, family history of premature CAD in first-degree relatives with male age less than 45 years or female age less than 55 years.

Study variables were defined as follows: hypertension: resting systolic blood pressure more than 140 mmHg, diastolic blood pressure more than 90 mmHg under standard procedures for blood pressure measurement, currently taking antihypertensive agents, or diagnosed as hypertension by physicians [6]; diabetes: meeting the diagnostic criteria by the American Diabetes Association, currently taking antidiabetic agents, or diagnosed as diabetes by physicians [7]; age gender variable identified by age over 45 in male or 55 in female; previous history of CAD identified by evidence of CAD by electrocardiography, echocardiography, radiographic study, or coronary angiogram.

Diagnosis of AMI was classified as ST elevation myocardial infarction (STEMI) and non-ST elevation myocardial infarction (Non-STEMI) according to the criteria provided by the American College of Cardiology Foundation/American Heart Association (ACCF/AHA) [8]. Unstable angina was also diagnosed by the ACCF/AHA guidelines. Patients with AMI or unstable angina were classified as ACS group, while patients with other causes were classified as non-ACS group.

The rate of ACS was calculated to evaluate the effectiveness of the nursing triage. Descriptive statistics were used to identify the differences between both groups. Variables with a *P* value less than 0.20 by univariate logistic regression or clinically significant variables such as age or gender were included in the subsequent multiple logistic regression analysis. The best model by multiple logistic regression analysis was the model with the highest adjusted *R* square. An online model predictive of ACS for nursing triage at the ED was produced from the best model by multiple logistic regression analysis. The online tool was available at http://chestpain-rama.webs.com/. The probability of having ACS ranged between 0 and 1.

## 3. Results

There were 175 patients who were identified by the nursing triage at the ED to be ACS at risk. Of those, 28 patients (16.0%) were diagnosed with ACS. The other 147 patients (84.0%) were diagnosed as non-ACS.

Clinical variables between ACS and non-ACS group were shown in Table 1. Those patients with ACS were significantly older (68 versus 64 years) and had higher proportions of age gender factor (96.43% versus 78.91%), diabetes (53.57% versus 21.09%), previous CAD (78.59% versus 40.82%), and presence of character of angina pain (75.00% versus 19.05%) than those patients without ACS.

Patients with diabetes and previous history of CAD or who had ACS chest pain were independently associated with having ACS by multivariate logistic regression (Table 2). The adjusted odds ratio (95% confidence interval) was 4.220 (1.445, 12.327), 3.333 (1.040, 10.684), and 12.539 (3.876, 40.567), respectively.

The online formula to predict risk of having ACS in patients presenting with chest pain at the ED was posted at http://chestpain-rama.webs.com/. The probability of having ACS was calculated based on the final model of multivariate logistic regression. The probability equals 1/[1 + *e* ^−*x*^]; *X* = −5.85 − [0.96 × gender] + [0.04 × age] + [1.20 × cad] + [1.44 × dm] + [2.53 × chest pain]; gender: female = 0 and male = 1; age: years; CAD: previous history of coronary artery disease; DM: no diabetes = 0 and diabetes = 1; chest pain: having one character of angina pain as mentioned in Methods.

## 4. Discussion

A crowded ED has been shown to be associated with poor cardiovascular outcomes in patients with chest pain at the ED [9]. High waiting time was significantly related to adverse outcomes in both patients with ACS and non-ACS. Nursing triage is one important step to correctly identify patients at risk for ACS [10, 11]. Unfortunately, only 16% of patients were identified correctly as having ACS by the nurse triage at the ED. This finding implied that nursing triage for ACS at the ED may need additional tool such as the online tool to improve the quality of the ACS nursing triage (http://chestpain-rama.webs.com/).

The present study showed that having diabetes mellitus and previous CAD and having characters of angina chest pain were significantly associated with having ACS in patients with chest pain at the ED (Table 2). These factors can be easily evaluated by nurses at the ED. Triage nurses can easily predict the risk of ACS by just filling out the online tool [5], hence leading to proper investigation and prompt treatment for the patients.

Factors in the tools are practical and are comprised of only clinical factors. Recently, some technology such as stress myocardial perfusion imaging or multislice computed tomography coronary angiography was used in the triage for chest pain patients to correctly diagnose ACS and also reduce hospitalization [12–14]. These investigations are expensive and not practical for nursing triage. The model in the present study however requires verification in a prospective fashion.

The Ramathibodi ACS nursing triage correctly identified ACS lower than that identified in a previous study from Spain (16.0% versus 21.7%) [15]. In Spain, the triage used flowchart as a tool to identify ACS. Instead of using flowchart, this study provided the online tool for nurse triage to evaluate the probability of having ACS. The significant predictors for ACS were mostly similar to the previous studies [15, 16]. Previous history of coronary intervention or CAD, history of diabetes, and characters of chest pain were also identified as risk factors for having CAD [15, 16].

Gender was included in the final model due to potential risk for ACS, but it did not declare the significant *P* value (Table 2). A previous study confirmed that there was no gender specific chest pain character for AMI [17]. Age was a significant factor for ACS by univariate logistic analysis; it was not an independent factor for ACS by multivariate logistic analysis though (Table 2). This finding implied that age is not an independent factor to identify ACS in patients with chest pain at the ED. The previous study showed that age less than 40 years was associated with non-ACS chest pain [5].

There are some limitations in this study. Due to retrospective study design, some risk factors for ACS were missing such as history of smoking or family history of sudden death. The total numbers of screened patients were also unable to be retrieved because the registration system recorded only final diagnosis. Data used in the study were obtained from the file of ACS nursing triage at the ED. Further studies should be performed to evaluate the validity of the triage online tool.

In conclusion, the effectiveness of the ED nurse triage for ACS was 16%. The online tool is available for the ED triage nurse to evaluate risk of ACS in individuals.

## Figures and Tables

**Table 1:** Features of patients presenting with chest pain at the emergency department categorized by having acute coronary syndrome (ACS).

Factors	No ACS *N* = 147	ACS *N* = 28	*P* value
Male gender	90 (61.22)	15 (53.57)	0.529
Median age (range), years	64 (12–93)	68 (44–88)	0.023
Age gender risk	116 (78.91)	27 (96.43)	0.031
Having diabetes mellitus	31 (21.09)	15 (53.57)	0.001
Previous CAD	60 (40.82)	22 (78.59)	<0.001
Having angina pain	28 (19.05)	21 (75.00)	<0.001

*Note.* Data presented as number (percentage) unless indicated otherwise; age gender risk indicated being male more than 45 years of age or female more than 55 years of age; CAD: coronary artery disease; angina pain indicated having at least one feature of typical angina pain including chest tightness; squeeze pain at retrosternum, referred to shoulder, arm, or mandible; dyspnea or tachypnea, associated with sweating or palpitation without other obvious causes, syncope or fainting, or improved with sublingual nitroglycerin.

**Table 2:** Factors associated with having acute coronary syndrome by logistic regression analysis.

Variables	Univariate odds ratio (95% confidence interval)	Adjusted odds ratio (95% confidence interval)
Male gender	0.731 (0.324, 1.648)	0.382 (0.117, 1.245)
Age	1.043 (1.006, 1.081)	1.036 (0.990, 1.084)
Having diabetes mellitus	4.318 (1.861, 10.019)	4.220 (1.445, 12.327)
Previous CAD	5.317 (20.34, 13.896)	3.333 (1.040, 10.684)
Having angina pain	12.750 (4.934, 32.945)	12.539 (3.876, 40.567)

*Note.* CAD: coronary artery disease; angina pain indicated having at least one feature of typical angina pain including chest tightness; squeeze pain at retrosternum, referred to shoulder, arm, or mandible; dyspnea or tachypnea, associated with sweating or palpitation without other obvious causes, syncope or fainting, or improved with sublingual nitroglycerin.
